# The relationship between albumin-corrected anion gap and hyperuricemia and its role in cardiovascular risk assessment: mediation effect analysis of triglycerides and non-high-density lipoproteins

**DOI:** 10.3389/fendo.2025.1668064

**Published:** 2025-09-10

**Authors:** Manli Yan, Wenhua Shi, Kaiyuan Zhang, Ping Gong, Hua Wei, Xiang Li

**Affiliations:** ^1^ Second Clinical Medical College, Guangzhou University of Chinese Medicine, Guangzhou, China; ^2^ Department of Endocrinology, Guangdong Provincial Hospital of Chinese Medicine, Guangzhou, China; ^3^ Department of Orthopedics, Guangdong Provincial Hospital of Chinese Medicine, Guangzhou, China

**Keywords:** serum albumin, anion gap, albumin-corrected anion gap, hyperuricemia, cardiovascular diseases

## Abstract

**Background:**

This study examines the relationship between Albumin-Corrected Anion Gap (ACAG) and hyperuricemia (HUA), as well as its potential mechanisms linking HUA to cardiovascular diseases. While HUA is associated with cardiovascular conditions, whether it is an independent risk factor remains unclear. ACAG, an indicator of acid-base balance, has prognostic value in cardiovascular outcomes, but its relationship with HUA has not been explored.

**Methods:**

Data from 4,588 adults who visited Guangdong Provincial Hospital of Chinese Medicine between January and December 2023 were analyzed. HUA was defined as a serum uric acid (SUA) level >420 μmol/L, with the remaining participants categorized as Non-HUA. SPSS, R, and “Zstats” software were used for data analysis.

**Results:**

Of the participants, 1,135 (24.7%) were in the HUA group, with 86.4% males. The ACAG, triglycerides (TG) and non-high-density lipoprotein cholesterol (non-HDL-C) levels in the HUA group were significantly higher than those in the Non-HUA group, while HDL-C levels were significantly lower. ACAG was positively correlated with SUA, even after adjusting for age and gender. Non-linear analysis showed that when ACAG exceeded 12.64, each additional unit increase was associated with a 12% increase in the adjusted odds ratio for HUA. Mediation analysis revealed that TG and non-HDL-C partially mediated the association between ACAG and HUA, accounting for 28.3% and 13.5%, respectively.

**Conclusion:**

This is the first study to demonstrate a significant correlation between ACAG and HUA, with TG and non-HDL-C acting as mediators, providing new directions for prevention and intervention of HUA.

## 1 Introduction

Hyperuricemia (HUA) (1) is a metabolic disorder characterized by an abnormal increase in serum uric acid (UA) levels. It can be triggered by various factors, including acquired factors (such as dietary habits, medication use, and bone marrow diseases) and genetic factors (such as enzyme deficiencies). In recent years, the incidence of HUA has been rising annually, with current data indicating that (2) it has become a prevalent metabolic disorder, second only to type 2 diabetes in prevalence, reaching 21.4% in the United States (2).

UA (1) is primarily synthesized in the liver, intestines and vascular endothelium. Due to its critical role in purine metabolism, UA is closely linked to various biological responses, including oxidative stress (3). Basic research has found that HUA exerts pathogenic effects on the occurrence and progression of cardiovascular diseases (4) through multiple pathways, including the renin-angiotensin system (1), nitric oxide synthase inhibition (5) and endothelial dysfunction (6). Additionally, HUA is considered an independent risk factor for cardiovascular and renal diseases (7), and is closely associated with hypertension (8), heart failure (9) and atherosclerotic heart disease (10). A U-shaped association has been identified between serum UA levels and all-cause mortality (3), indicating that both excessively high (3) and low (11) UA concentrations are linked to adverse outcomes. This presents challenges for the clinical management of hyperuricemia. These findings suggest that optimal regulation of serum UA may play an important role in the prevention and management of cardiovascular diseases. Numerous studies (12) have confirmed the clinical benefits of enhanced UA management; however, it remains unclear whether serum UA is merely a contributing factor to cardiovascular disease or a pathogenic risk factor and potential therapeutic target in clinical practice.

The anion gap (AG) is a commonly used biochemical marker in the clinical assessment of acid-base balance, and it is considered valuable for evaluating disease severity and predicting prognosis. In practice, the measurement of AG can be influenced by various factors. However, the novel biochemical marker known as the “albumin-corrected anion gap” (ACAG) can more effectively minimize these interferences.

Previous studies have shown that ACAG is closely associated with the prognosis of patients with acute myocardial infarction (13), including 30-day all-cause mortality (14). It has also been linked to poor in-hospital outcomes in cardiac arrest patients (15), as well as in-hospital and long-term mortality in patients undergoing coronary artery bypass grafting (16). Furthermore, ACAG has been associated with the prognosis of patients with aneurysmal subarachnoid hemorrhage (17).

However, there have been no studies examining the correlation between ACAG and HUA to date. Therefore, we collected baseline information from adult participants using the health examination system at Guangdong Provincial Hospital of Chinese Medicine. This cross-sectional study aimed to explore the relationship between HUA and ACAG, and through big data analysis, to further investigate the potential association between HUA and cardiovascular diseases.

## 2 Materials and methods

### 2.1 Data collection

We extracted data from adult participants who visited Guangdong Provincial Hospital of Chinese Medicine from January 2023 to December 2023 and had complete data. After assessing the availability of laboratory data, a total of 4,588 participants were included in the study (2,276 males and 2,312 females). According to the “Chinese Guidelines for the Diagnosis and Treatment of Hyperuricemia and Gout (2019)” (18), a serum UA level greater than 420 μmol/L was defined as HUA, while the remaining participants were categorized as the Non-HUA group.

### 2.2 Statistical analysis

We summarized the baseline characteristics of participants with HUA and Non-HUA. Categorical variables were expressed as percentages and compared between groups using the chi-square test. The distribution of continuous variables was assessed using the Kolmogorov-Smirnov test. Since all continuous variables in this study did not follow a normal distribution, these variables were presented as median [interquartile range], and differences between groups were evaluated using the Mann-Whitney U test.

To investigate the nonlinear relationship between the ACAG index and UA, as well as HUA, we employed the restricted cubic spline (RCS) model. Additionally, linear regression analysis was conducted to examine the relationship between the ACAG index and UA, while univariate and multivariate logistic regression analyses were performed to assess the association between the ACAG index and HUA.

We constructed three different regression models: an unadjusted model, Model 1 (adjusting for sex and age), and Model 2 (further adjusting for sex, age, TG and non-HDL-C). The ACAG index was transformed into a categorical variable based on tertiles to calculate trend P-values, verifying the consistency of the ACAG index as a continuous variable with the categorical variable. Given the nonlinear relationship between ACAG and HUA, we identified the inflection point using RCS and conducted threshold effect analysis with piecewise logistic regression models to further explore the relationship between the two.

To determine whether the effect of the ACAG index on HUA is influenced by lipid levels (TG and non-HDL-C), we conducted a mediation analysis. This analysis quantified the total effect (the association between ACAG and HUA), the direct effect (the total effect not influenced by lipid levels), and the indirect effect (the influence of ACAG on HUA attributed to lipids). The mediation analysis adjusted for sex and age. Mediation analyses were performed using the “mediation” package in R.

All statistical tests were two-tailed, with a p-value of less than 0.05 considered statistically significant. Data analyses were performed using SPSS software (version 26.0), R software (version 4.2.1) and “Zstats.”

## 3 Results

### 3.1 Baseline characteristics of study participants

A total of 4588 participants were included in this study, with baseline characteristics presented in **Table 1**. The median age was 47.0 years (IQR 39.0, 55.0), and females accounted for 50.4% of the participants. Among these, 1,135 individuals were classified into the HUA group, representing 24.7% of the total cohort. Compared to the non-HUA group, the HUA group had a significantly higher proportion of males, as well as elevated levels of ACAG, TG, and non-HDL-C. Conversely, the level of HDL-C was markedly lower in the HUA group.

**Table 1:** Comparison of baseline characteristics of adult participants.

Variables	Total (n= 4588)	Non-HUA group (n= 3453)	HUA group (n= 1135)	*P-*value
Age (median (IQR))	47.0 (39.0, 55.0)	47.00 [39.00, 55.00]	46.00 [37.00, 54.00]	<0.001
Sex (%)				<0.001
Male	49.6%	37.5%	86.4%	
Female	50.4%	62.5%	13.6%	
WBC (10^9/L) (median (IQR))	5.89 [5.01, 6.96]	5.75 [4.87, 6.80]	6.41 [5.47, 7.36]	<0.001
NEU (10^9/L) (median (IQR))	3.34 [2.69, 4.10]	3.27 [2.63, 4.03]	3.56 [2.92, 4.26]	<0.001
LYM (10^9/L) (median (IQR))	1.95 [1.62, 2.34]	1.90 [1.57, 2.28]	2.12 [1.79, 2.51]	<0.001
MONO (10^9/L) (median (IQR))	0.35 [0.27, 0.43]	0.33 [0.26, 0.41]	0.39 [0.32, 0.47]	<0.001
RBC (10^9/L) (median (IQR))	4.81 [4.46, 5.19]	4.71 [4.40, 5.07]	5.13 [4.83, 5.39]	<0.001
Hb (g/L) (median (IQR))	143.00 [132.00, 155.00]	139.00 [129.00, 151.00]	153.00 [144.00, 161.00]	<0.001
PLT (10^9/L) (median (IQR))	245.00 [211.00, 282.00]	246.00 [212.00, 282.00]	245.00 [211.00, 281.00]	0.634
TG (mmol/L) (median [IQR])	1.23 [0.90, 1.83]	1.13 [0.84, 1.63]	1.71 [1.22, 2.49]	<0.001
TC (mmol/L) (median [IQR])	5.14 [4.54, 5.82]	5.08 [4.49, 5.76]	5.28 [4.70, 5.96]	<0.001
HDL-C (mmol/L) (median [IQR])	1.34 [1.13, 1.61]	1.42 [1.19, 1.68]	1.18 [1.02, 1.36]	<0.001
nonHDL-C (mmol/L) (median [IQR])	3.75 [3.11, 4.41]	3.63 [3.01, 4.31]	4.09 [3.51, 4.72]	<0.001
LDL-C (mmol/L) (median [IQR])	3.25 [2.67, 3.83]	3.21 [2.62, 3.79]	3.38 [2.83, 3.92]	<0.001
Apo-A1 (median [IQR])	1.56 [1.39, 1.74]	1.59 [1.42, 1.77]	1.47 [1.33, 1.60]	<0.001
Apo-B (median [IQR])	1.02 [0.84, 1.20]	0.99 [0.82, 1.17]	1.12 [0.95, 1.28]	<0.001
ALB (g/L) (median (IQR))	46.20 [44.50, 47.90]	46.00 [44.20, 47.60]	46.90 [45.30, 48.60]	<0.001
AG (mmol/L) (median (IQR))	13.20 [11.20, 15.10]	13.10 [11.10, 14.90]	13.60 [11.60, 15.70]	<0.001
ACAG (median (IQR))	12.64 [10.62, 14.68]	12.57 [10.57, 14.50]	12.93 [10.86, 15.12]	<0.001
UA (μmol/L) (median (IQR))	350.00 [288.00, 419.25]	319.00 [270.00, 366.00]	471.00 [443.00, 515.00]	<0.001

### 3.2 RCS analysis of the relationship between ACAG and UA and HUA

**Figure 1:**
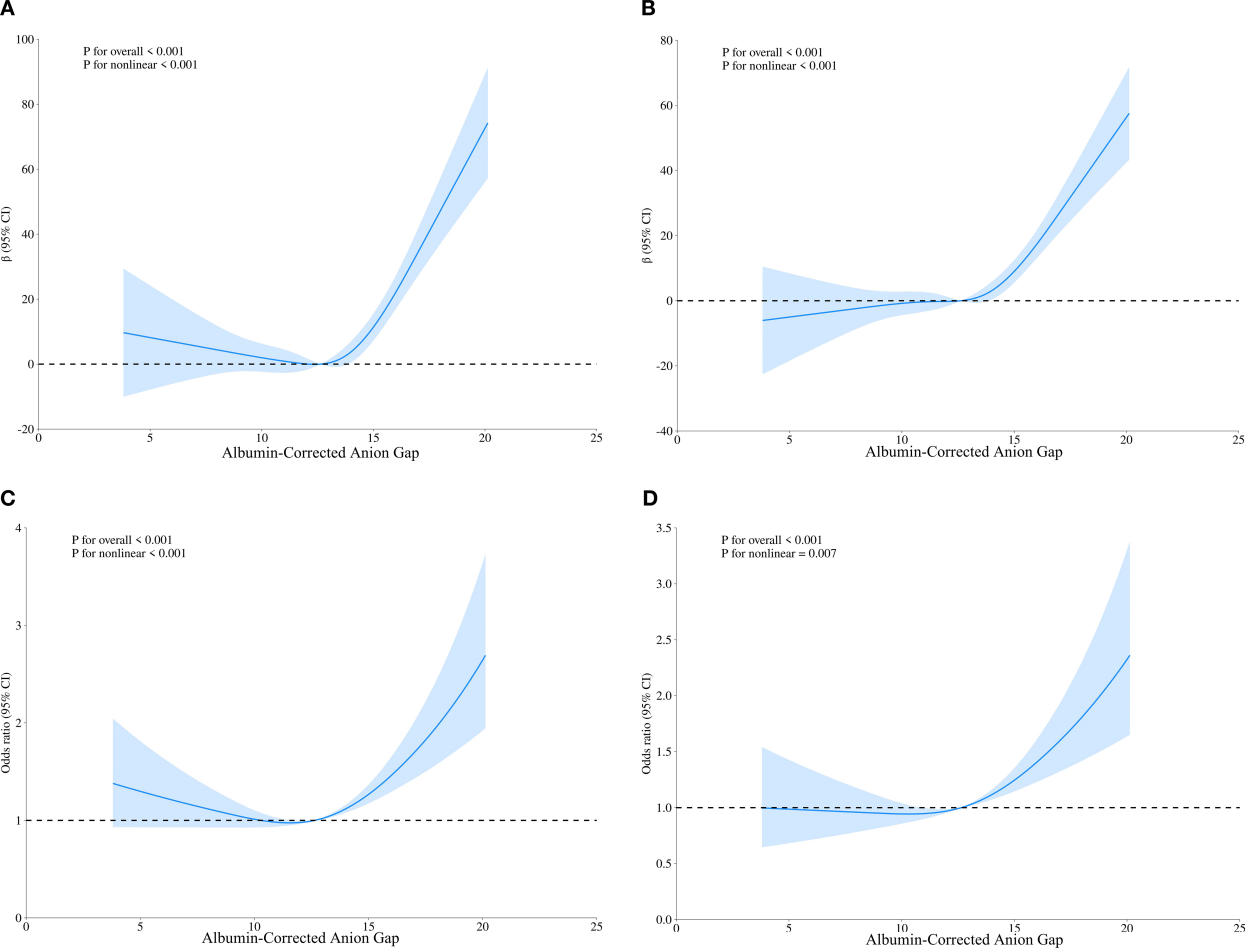
Restricted cubic spline regression analysis of ACAG with UA levels and HUA. **(A)** Sample curves are for the relationship between ACAG and UA levels, **(C)** Sample curves are for the relationship between ACAG and HUA; **(B, D)** Sample curves adjusted for age and gender.

We employed RCS analysis to reveal the nonlinear relationships between the ACAG, UA and HUA(P for nonlinear <0.001) (**Figures 1A, C**). The results demonstrated a significant positive correlation between elevated ACAG and UA levels (β=3.10, 95% CI, 2.15-4.06), which remained significant after adjusting for gender and age (Adjusted β=3.03, 95% CI, 2.24-3.83; **Figure 1B**, **Table 2**). Additionally, after adjusting for TG and non-HDL-C, the positive correlation between ACAG and UA levels was consistent with the initial model and Model 1.

**Table 2:** Relationship between ACAG, UA and HUA.

Variables	Crude	Model 1	Model 2
	β (95%CI)	β (95%CI)	β (95%CI)
ACAG level	3.10 (2.15 ~ 4.06)	3.03 (2.24 ~ 3.83)	2.32 (1.54 ~ 3.09)
	OR (95%CI)	OR (95%CI)	OR (95%CI)
ACAG level	1.05 (1.03 ~ 1.08)	1.06 (1.03 ~ 1.09)	1.04 (1.02 ~ 1.07)
ACAG subgroups			
Tertile 1	Ref.	Ref.	Ref.
Tertile 2	1.01 (0.85 ~ 1.19)	1.07 (0.89 ~ 1.29)	1.06 (0.88 ~ 1.28)
Tertile 3	1.34 (1.13 ~ 1.57)	1.44 (1.20 ~ 1.72)	1.31 (1.09 ~ 1.58)
*P* for trend	<0.001	<0.001	0.005

OR, Odds Ratio; CI, Confidence Interval.

Crude: unadjusted.

Model 1: adjusted for age, sex.

Model 2: adjusted for age, sex, TG, Non-HDL-C.

In all three models, ACAG was closely associated with HUA, both as a continuous and categorical variable (**Table 2**). Compared to the first tertile of ACAG, the third tertile showed a positive correlation with HUA (Crude model: OR, 1.34, 95% CI, 1.13 - 1.57; Model 1: OR, 1.44, 95% CI, 1.20 - 1.72; Model 2: OR, 1.31, 95% CI, 1.09 - 1.58). Trend tests further confirmed that increased ACAG was associated with HUA (P for trend <0.05).

### 3.3 Threshold effect analysis for ACAG and HUA

After adjusting for gender and age, the RCS analysis showed that ACAG and HUA still had a significant nonlinear relationship (P for nonlinear < 0.001) (**Figure 1D**). Based on the RCS analysis we obtained an inflection point (ACAG = 12.64) and built a two-stage logistic regression model, which revealed that when ACAG was lower than 12.64, there was no significant increase in the adjusted OR for hyperuricemia for each 1-unit increase in ACAG. When ACAG exceeded 12.64, the adjusted OR for hyperuricemia increased by 12% (Adjusted OR 1.12, 95% CI 1.07, 1.18) for each 1-unit increase in ACAG (**Table 3**).

**Table 3:** Threshold effect analysis between ACAG and HUA.

Outcome	Adjusted OR (95% CI)	*P*-value
Model 1 Fitting by standard linear model	1.06 (1.03-1.09)	<0.001
Model 2 Fitting by two-piecewise linear model		
Inflection point	12.64	
ACAG < 12.64	1.00 (0.96-1.05)	0.870
ACAG ≥ 12.64	1.12 (1.07-1.18)	<0.001
*P* for log-likelihood ratio	0.012	

Adjusted: age and sex.

### 3.4 Mediation of ACAG-HUA correlation by TG and non-HDL-C

We found that ACAG, TG and non-HDL-C were positively correlated with HUA. A positive correlation between ACAG and TG and non-HDL-C was also demonstrated during data analysis. This suggests that TG and non-HDL-C may be the link between ACAG and HUA by a potential mechanism. (See **Supplementary Tables 1**-**3**) Therefore, to further explore the roles of TG, non-HDL-C in mediating the association between ACAG and HUA, we performed mediation analyses to examine their internal relationships.

**Figure 2:**
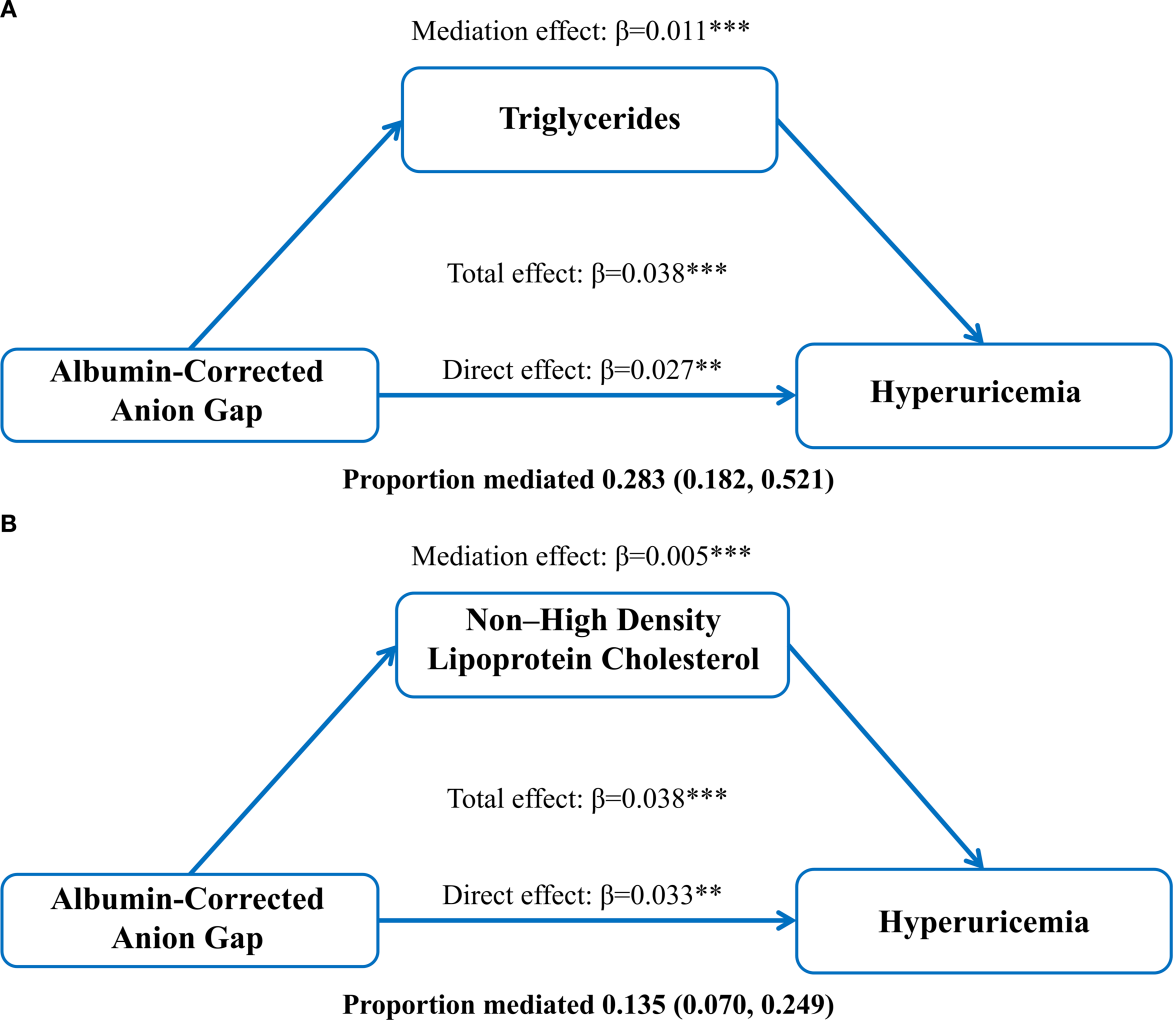
Mediation of the association between albumin-corrected anion gap and hyperuricemia by triglycerides **(A)** and non-high-density lipoprotein **(B)**, adjusted for age and gender. **P < 0.01, ***P < 0.001.

As shown in **Figure 2**, ACAG had a significant direct effect on HUA (β= 0.027, 95% CI: 0.011, 0.042), TG partially mediated the indirect effect of ACAG on HUA (β=0.011 95% CI: 0.008, 0.014), and non-HDL-C partially mediated the indirect effect of ACAG on HUA (β= 0.005 95% CI: 0.003, 0.008). Approximately 28.3% and 13.5% of the effects of ACAG on HUA were mediated through TG and non-HDL-C levels, respectively.

## 4 Discussion

In healthy adults, the liver produces approximately 0.2 g/kg of serum albumin (ALB) daily, based on body weight (19). ALB is the most abundant protein in human serum, accounting for about 60-65% of all proteins (19, 20). In addition to maintaining colloid osmotic pressure, ALB serves as a carrier in the serum through various post-translational modifications, such as glycation, oxidation, partial loss, and carbamylation (20).

Reversible modifications of proteins are crucial for preventing irreversible damage and regulating protein function (21). Moreover, cysteine-34 (Cys-34) (21) is the only cysteine residue in ALB that exists in a free form. Research suggests that (21) the antioxidant properties of ALB largely depend on Cys-34 and its role in maintaining vascular homeostasis, including the protection of vascular endothelium under conditions associated with oxidative stress. Therefore, ALB is regarded as one of the body’s important antioxidants, with its relative contribution reported to range from approximately 40% to 70% of total antioxidant capacity (22).

Reactive oxygen species (ROS) play a significant pathogenic role in the development of various systemic diseases. The inherent nature of oxidative stress arises from an imbalance between the production and degradation of ROS, which involves multiple pathological processes across different systems. Proteins are critical targets for ROS, as they can suffer irreversible damage that impairs their physiological functions, leading to issues such as cardiac cell injury (22).

Studies (23–25) have shown that UA plays a role in promoting the elimination of reactive ROS, acting as an antioxidant capable of scavenging up to 55% of extracellular free radicals. However, the process of UA formation is also accompanied by the generation of ROS. When extracellular uric acid reacts with myeloperoxidase, it produces a compound with pro-oxidative properties known as hydrogen peroxide urate. In cases of HUA, the increased concentration of extracellular uric acid enhances this reaction. Consequently, HUA may induce oxidative stress, which can, in turn, promote inflammation.

Our study found that the ALB levels in the HUA group were significantly higher than those in the Non-HUA group (P<0.001). This observation, when combined with previous research, suggests that the elevated ALB levels in HUA patients may be closely linked to a heightened inflammatory state and the body’s response to oxidative stress. Higher ALB levels could reflect a pathological compensation for a stronger inflammatory burden in the body; however, this hypothesis requires further experimental validation.

The AG represents the difference between the concentrations of negatively charged anions (13) and positively charged cations in plasma, primarily consisting of negatively charged ALB (26). It is primarily used to assess acid-base balance and electrolyte disturbances. Our study indicates that the AG levels in the HUA group were significantly higher than those in the Non-HUA group (P<0.001). High AG metabolic acidosis is a subclass of metabolic acidosis (27), consistent with the traditional understanding that the pH in HUA patients tends to be acidic.

Acid retention (28) can reduce the pH levels in renal interstitium and intracellular compartments, leading to increased levels of angiotensin II, aldosterone, endothelin, and pro-inflammatory cytokines in the kidneys—all of which are associated with renal fibrosis and damage. Research has shown (26, 29) that the excretion of UA increases with rising pH levels in the renal tubule. Clinically, physicians often use sodium bicarbonate to alkalize urine and adjust dietary acid-base balance to influence the pH-dependent transport systems in the kidneys, thereby affecting UA excretion. Additionally, individuals who consume animal proteins are more prone to HUA compared to vegetarians (29), which may also be a significant factor contributing to the higher ALB levels observed in the HUA group compared to the Non-HUA group.

Compared to using ALB and AG individually, the ACAG provides a more precise assessment of hidden tissue anions, allows for a better assessment of acid-base homeostasis, and partially reflects nutritional status. Gao et al. (26) conducted a retrospective analysis of clinical data from 9,625 critically ill patients with acute kidney injury (AKI), revealing that elevated ACAG levels (greater than 20 mmol/L) upon ICU admission significantly increased the 30-day and 360-day all-cause mortality rates. Similarly, Zhao et al. (28) identified a correlation between higher ACAG levels and the risk of AKI, suggesting that ACAG may serve as an early indicator of adverse outcomes in ICU patients.

A key advantage of this approach lies in its ability to not only highlight abnormalities in acid-base balance but also potentially reflect underlying malnutrition, providing a more comprehensive assessment tool for clinicians. In our study, we used RCS analysis to reveal a nonlinear relationship between ACAG, UA and HUA (P for nonlinear <0.001). The increase in ACAG showed a significant positive correlation with UA levels (β=3.10, 95% CI, 2.15-4.06), and this significance remained after adjusting for sex and age. When ACAG exceeded 12.64, each additional unit of ACAG was associated with a 12% increase in the odds ratio for HUA.

These findings suggest that monitoring ACAG levels may aid in predicting and managing HUA and its related complications. This evidence provides important clinical insights and emphasizes the critical role of acid-base balance monitoring in evaluating patients’ metabolic status.

In clinical practice, it is frequently observed that HUA is associated with an increased risk of cardiovascular and cerebrovascular diseases. Previous studies have indicated a close relationship between the ACAG and the occurrence and progression of cardiovascular diseases, and our data also suggest its role in HUA. However, there is currently a lack of in-depth exploration regarding the mediating relationship between HUA and ACAG. Therefore, we conducted a more detailed study that integrates clinical realities.

Hypertriglyceridemia (30) is a disorder of lipoprotein metabolism and is recognized as a significant risk factor for cardiovascular diseases. A retrospective analysis by Hou et al. (30) involving 3,884 subjects found that TG are an independent risk factor for HUA, with increasing levels of TG correlated with a heightened risk of HUA. Notably, there are significant differences in glycerolipid metabolism (31) between HUA patients and the normal population; the conversion of certain metabolites, such as saturated fatty acids, is associated with the production of inflammatory factors, which may contribute to the increased inflammatory burden in HUA patients.

While LDL-C has traditionally been the primary focus in the treatment of dyslipidemia, non-HDL-C (32) has increasingly been recognized as an important risk factor for cardiovascular diseases and is gradually becoming a key target in the management of lipid abnormalities. A retrospective analysis by Xu et al. (33)involving 9,580 subjects demonstrated a significant association between non-HDL-C and HUA, suggesting the need for future development of therapies that can simultaneously lower both triglyceride and UA levels to manage the combined syndrome of hyperuricemia and hyperlipidemia.

Through data analysis, we found a significant direct association between ACAG and HUA (β = 0.027, 95% CI: 0.011, 0.042). Additionally, TG partially accounted for the relationship between ACAG and HUA (β = 0.011, 95% CI: 0.008, 0.014), while non-HDL-C also explained part of this association (β = 0.005, 95% CI: 0.003, 0.008). Approximately 28.3% and 13.5% of the relationship between ACAG and HUA was explained by TG and non-HDL-C levels, respectively, which is consistent with previous research findings.

In this study, although we achieved several important findings, several limitations should be acknowledged. The foremost limitation is the cross-sectional nature of our design, which fundamentally prevents the establishment of causality or temporality between the observed variables. This inherent constraint of the cross-sectional approach means that our findings can only be interpreted as associations rather than evidence of cause and effect. Furthermore, the relatively small sample size and single-center setting may limit the generalizability of our results, as regional variations in diet, metabolism, and lifestyle could influence HUA. Second, although we analyzed various biochemical markers and adjusted for key variables, other potential confounding factors—such as genetic background, dietary patterns, medication use, renal function, inflammatory status, and environmental exposures—were not fully accounted for, which might introduce bias into the observed associations. Additionally, the use of static laboratory values may not fully capture the dynamic metabolic state of the patients. Therefore, future research should prioritize multicenter, large-scale prospective cohort studies to overcome these limitations. Such longitudinal designs are essential to establish temporality, control for unmeasured confounders, and validate the associations found here. The implementation of dynamic monitoring methods could also provide a more comprehensive metabolic perspective. Furthermore, while our research preliminarily explored the relationships among ACAG, TG, and non-HDL-C, more in-depth mechanistic studies are needed to validate and elucidate these associations. Finally, before any potential clinical applications of our findings can be implemented, their safety and effectiveness must be rigorously validated through interventional studies.

## 5 Conclusion

The findings of this present study demonstrate that patients with HUA exhibit significantly higher levels of ALB and AG than those without HUA, suggesting a heightened inflammatory state and possible compensatory mechanisms in this population. Furthermore, a positive correlation was identified between ACAG and UA levels. Further analysis revealed that TG and non-HDL-C partially mediate the association between ACAG and HUA, underscoring the pivotal role of lipid metabolism disturbances in the development of HUA.

These results provide valuable clinical implications and generate important hypotheses, supporting the potential utility of monitoring acid-base balance and related metabolic biomarkers in the prediction and management of HUA and its associated complications. Future investigations should prioritize longitudinal or interventional designs aimed at elucidating the underlying mechanisms of these biomarkers and validating their applications in risk stratification and tailored interventions. Such research is essential to contribute to more effective strategies for the prevention and treatment of hyperuricemia.

## Data Availability

The original contributions presented in the study are included in the article/**Supplementary Material**. Further inquiries can be directed to the corresponding authors.

## Supplementary material

Supplementary Table 1 Relationship between TG, non-HDL-C, and UA levels. OR, Odds Ratio; CI, Confidence Interval; Crude, unadjusted. Model 1: adjusted for age, sex. Model 2: adjusted for age, sex, TG, non-HDL-C.

Supplementary Table 2 Relationship between ACAG and TG. OR, Odds Ratio; CI, Confidence Interval; Crude: unadjusted. Model 1: adjusted for age, sex. Model 2: adjusted for age, sex, TG, non-HDL-C.

Supplementary Table 3 Association between ACAG and non-HDL-C. OR, Odds Ratio; CI, Confidence Interval; Crude: unadjusted. Model 1: adjusted for age, sex. Model 2: adjusted for age, sex, TG, non-HDL-C.
