# Supplementary material for: The relationship between albumin-corrected anion gap and hyperuricemia and its role in cardiovascular risk assessment: mediation effect analysis of triglycerides and non-high-density lipoproteins
Source: Front Endocrinol (Lausanne). 2025 Sep 10;16:1668064. doi: 10.3389/fendo.2025.1668064 (PMC12457168; doi:10.3389/fendo.2025.1668064)
Supplement: Supplementary Table 1 — Relationship between TG, non-HDL-C, and UA levels. OR, Odds Ratio; CI, Confidence Interval; Crude, unadjusted. Model 1: adjusted for age, sex. Model 2: adjusted for age, sex, TG, non-HDL-C. [file Table1.docx]

| Variable | Crude | Model 1 | Model 2 |
| --- | --- | --- | --- |
|  | β (95%CI) | β (95%CI) | β (95%CI) |
| TG | 14.91 (13.32 ~ 16.50) | 9.33 (7.95 ~ 10.71) | 5.99 (4.52 ~ 7.46) |
| Non-HDL-C | 24.64 (22.09 ~ 27.19) | 17.81 (15.60 ~ 20.03) | 14.10 (11.72 ~ 16.49) |

**Supplementary Table 1 Relationship Between TG, non-HDL-C, and UA Levels**

OR: Odds Ratio; CI: Confidence Interval;

Crude: unadjusted.

Model 1: adjusted for age, sex.

Model 2: adjusted for age, sex, TG, non-HDL-C.

**Supplementary Table 2 Relationship between ACAG and TG**

| Variables | Crude | Model 1 | Model 2 |
| --- | --- | --- | --- |
|  | β (95%CI) | β (95%CI) | β (95%CI) |
| ACAG level | 0.07 (0.06 ~ 0.09) | 0.07 (0.05 ~ 0.09) | 0.06 (0.04 ~ 0.07) |
| ACAG subgroups |  |  |  |
| Tertile 1 | Ref. | Ref. | Ref. |
| Tertile 2 | 0.03 (-0.09 ~ 0.14) | 0.03 (-0.08 ~ 0.15) | 0.02 (-0.09 ~ 0.13) |
| Tertile 3 | 0.42 (0.30 ~ 0.53) | 0.41 (0.29 ~ 0.52) | 0.34 (0.24 ~ 0.45) |
| *P* for trend | <0.001 | <0.001 | <0.001 |

OR: Odds Ratio; CI: Confidence Interval;

Crude: unadjusted.

Model 1: adjusted for age, sex.

Model 2: adjusted for age, sex, TG, non-HDL-C

**Supplementary Table 3 Association between ACAG and non-HDL-C**

| Variable | Crude | Model 1 | Model 2 |
| --- | --- | --- | --- |
|  | β (95%CI) | β (95%CI) | β (95%CI) |
| ACAG level | 0.03 (0.02 ~ 0.04) | 0.02 (0.01 ~ 0.03) | 0.01 (-0.00 ~ 0.02) |
| ACAG subgroups |  |  |  |
| Tertile 1 | Ref. | Ref. | Ref. |
| Tertile 2 | 0.03 (-0.05 ~ 0.10) | 0.02 (-0.05 ~ 0.09) | 0.01 (-0.05 ~ 0.08) |
| Tertile 3 | 0.12 (0.05 ~ 0.20) | 0.11 (0.04 ~ 0.18) | 0.01 (-0.06 ~ 0.08) |
| *P* for trend | <0.001 | 0.003 | 0.749 |

OR: Odds Ratio; CI: Confidence Interval;

Crude: unadjusted.

Model 1: adjusted for age, sex.

Model 2: adjusted for age, sex, TG, non-HDL-C.
